# 

**Published:** 1900-09

**Authors:** 


SEPTEMBER, 1900.

*Vol. XVIII.* *No.* **69.**

LIBRARY [illegible] 30.

### THE BRISTOL MEDICO-CHIRURGICAL JOURNAL

A Quarterly Journal of the Medical Sciences for the West of England and South Wales

*PUBLISHED UNDER THE AUSPICES OF*
*THE BRISTOL MEDICO-CHIRURGICAL SOCIETY.*

LIBRARY
SURGEON GENERAL'S OFFICE
OCT.-14.-1900

"Scire est nescire, nisi id me

Scire alius sciret."

*Editor:* R. SHINGLETON SMITH, M.D., B.Sc.
WITH WHOM ARE ASSOCIATED
J. MICHELL CLARKE, M.A., M.D.,
J. LACY FIRTH, M.S., M.D., AND JAMES SWAIN, M.S., M.D.
*Assistant-Editor:* P. WATSON WILLIAMS, M.D.
*Editorial Secretary:* JAMES TAYLOR.

BRISTOL: J. W. ARROWSMITH.
LONDON: J. & A. CHURCHILL, 7 GREAT MARLBOROUGH STREET.

*Price One Shilling and Sixpence.*

